# *RHO* Mutations (p.W126L and p.A346P) in Two Japanese Families with Autosomal Dominant Retinitis Pigmentosa

**DOI:** 10.1155/2014/210947

**Published:** 2014-11-16

**Authors:** Satoshi Katagiri, Takaaki Hayashi, Masakazu Akahori, Takeshi Itabashi, Jo Nishino, Kazutoshi Yoshitake, Masaaki Furuno, Kazuho Ikeo, Tetsuji Okada, Hiroshi Tsuneoka, Takeshi Iwata

**Affiliations:** ^1^Division of Molecular and Cellular Biology, National Institute of Sensory Organs, National Hospital Organization Tokyo Medical Center, 2-5-1 Higashigaoka, Meguro-ku, Tokyo 152-8902, Japan; ^2^Department of Ophthalmology, The Jikei University School of Medicine, 3-25-8 Nishi-shimbashi, Minato-ku, Tokyo 105-8461, Japan; ^3^Department of Life Science, Gakushuin University, 1-5-1 Mejiro, Toshima-ku, Tokyo 171-8588, Japan; ^4^Laboratory of DNA Data Analysis, National Institute of Genetics, 1111 Yata Mishima, Shizuoka 411-8540, Japan; ^5^RIKEN Center for Life Science Technologies, Division of Genomic Technologies, Life Science Accelerator Technology Group, Transcriptome Technology Team, 1-7-22 Suehiro-cho, Tsurumi-ku, Yokohama, Kanagawa 230-0045, Japan

## Abstract

*Purpose*. To investigate genetic and clinical features of patients with rhodopsin (*RHO*) mutations in two Japanese families with autosomal dominant retinitis pigmentosa (adRP). *Methods*. Whole-exome sequence analysis was performed in ten adRP families. Identified *RHO* mutations for the cosegregation analysis were confirmed by Sanger sequencing. Ophthalmic examinations were performed to evaluate the RP phenotypes. The impact of the *RHO* mutation on the rhodopsin conformation was examined by molecular modeling analysis. *Results*. In two adRP families, we identified two *RHO* mutations (c.377G>T (p.W126L) and c.1036G>C (p.A346P)), one of which was novel. Complete cosegregation was confirmed for each mutation exhibiting the RP phenotype in both families. Molecular modeling predicted that the novel mutation (p.W126L) might impair rhodopsin function by affecting its conformational transition in the light-adapted form. Clinical phenotypes showed that patients with p.W126L exhibited sector RP, whereas patients with p.A346P exhibited classic RP. *Conclusions*. Our findings demonstrated that the novel mutation (p.W126L) may be associated with the phenotype of sector RP. Identification of *RHO* mutations is a very useful tool for predicting disease severity and providing precise genetic counseling.

## 1. Introduction

Retinitis pigmentosa (RP) is a heterogeneous group of inherited retinal disorders characterized by night blindness, constricted visual fields, abnormal color vision, and retinal degeneration. The prevalence of RP is approximately 1 per 4000 persons, with more than 1 million affected individuals existing worldwide [1]. RP patients show various inheritance patterns including autosomal recessive, autosomal dominant, X-linked, mitochondrial [2], and digenic [3] inheritance.

Autosomal dominant RP (adRP) makes up 30 to 40% of the overall RP cases, while mutations in the rhodopsin (*RHO*) gene are responsible for about 25% of adRP cases found in Caucasians [1]. The* RHO* gene has been mapped to the long arm of chromosome 3 (3p21-24) and encodes 348 amino acids [4]. In 1990, the* RHO* gene was first described in the literature as being the causative gene for adRP [5, 6]. While rhodopsin is a typical seven transmembrane G-protein-coupled receptor, a photon and not a molecular ligand is responsible for initiating the rhodopsin phototransduction cascade. When rhodopsin absorbs the photon, retinal chromophore (11-*cis*-retinal) changes to all-*trans*-retinal. The conformational changes that occur in rhodopsin result in the hyperpolarization of the rod cells, which play an important role in vision [7].

Dominant (or heterozygous)* RHO* mutations have been reported to show two different RP phenotypes, classic RP and sector RP [8–12]. Classic RP is a typical form of RP that is characterized by early-onset and diffuse/generalized retinal dysfunction, whereas sector RP is characterized by adult-onset and regionalized/sectorial retinal dysfunction [13–15]. Sector RP, as originally described by Bietti in 1937 [16], is characterized by retinal degeneration that is limited to one or two quadrants of the fundus and slowly progression compared with classic RP [13–15].

The frequency of the* RHO* mutations in adRP differs between ethnic groups. For example, there is a much lower frequency of* RHO* mutations in Japanese, Chinese, and Korean populations compared with European populations [17–19]. Saga et al. found* RHO* mutations in 1/13 (7.7%) adRP Japanese patients [20], whereas a separate study found that 43/150 (29%) adRP patients in North America had the* RHO* mutations [21]. Thus,* RHO* mutations have not been considered a major cause of adRP in Japanese patients.

To date, only a small number of* RHO* mutations have been reported in the Japanese population [20, 22–25]. In our current study, we used a whole-exome sequencing technique and identified two* RHO* mutations in two Japanese families with adRP, one (p.W126L) of which was novel. We additionally examined the impact of the p.W126L mutation on rhodopsin conformation by investigating the molecular modeling.

## 2. Material and Methods

The protocol of this study was approved by the Institutional Research Board of The Jikei University School of Medicine and National Hospital Organization Tokyo Medical Center. The protocol adhered to the tenets of the Declaration of Helsinki, and informed consent was obtained from all participants.

### 2.1. Clinical Studies

**Figure 1:**
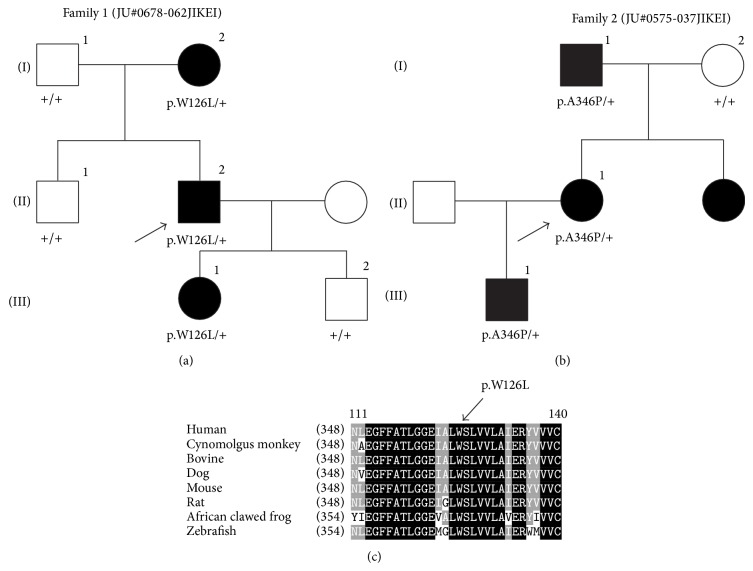
Pedigrees of the two Japanese families with RP and amino acid sequence alignment of the rhodopsin in different vertebrate species. (a) and (b) The solid squares (male) and circles (female) represent the affected individuals. The proband of each family is indicated by the arrows. (c) The tryptophan residue at position 126 is highly conserved. The conserved amino acids between the different species are shown in the black boxes. The less and least conserved amino acids are highlighted in the gray or white boxes, respectively.

Ten unrelated adRP patients from ten Japanese families were recruited for this study. RP diagnosis was based on the visual field, fundus examination, and electroretinogram (ERG) findings. Detailed ophthalmic examinations were conducted in the two families that exhibited the* RHO* mutations (family 1: JU#0678-062JIKEI and family 2: JU#0575-037JIKEI) (Figures 1(a) and 1(b)). These evaluations included decimal best-corrected visual acuity (BCVA), slit-lamp, and fundus examinations, optical coherence tomography (OCT) (Cirrus HD-OCT; Carl Zeiss Meditec AG, Dublin, CA), and fundus autofluorescence imaging (Spectralis HRA; Heidelberg Engineering, Heidelberg, Germany). Visual fields were assessed with kinetic Goldmann perimetry (GP; Haag Streit, Bern, Switzerland). Full-field ERG was performed according to the protocols of the International Society for Clinical Electrophysiology of Vision. Details of the methods and normal data have been reported previously [26].

### 2.2. DNA Preparation and Exome Sequencing Analysis

After obtaining venous blood samples from ten adRP patients, genomic DNA was extracted. Whole-exome sequencing was performed in all ten adRP patients using a previously described method [27]. The obtained sequence data in the patients were compared with reference human genome sequences (1000 genomes phase 2 reference, hs37d5). Subsequently, we then focused on only the variants that could change the amino acid sequence, such as the nonsynonymous variants, splice acceptor, and donor site variants, and the short insertions and deletions. In the next step, we filtered the remaining variants using the criteria that the frequency of the variant had to be less than 1% in the databases of the 1000 Genomes project (http://www.1000genomes.org) and the Human Genetic Variation Browser (http://www.genome.med.kyoto-u.ac.jp/SnpDB/index.html). In the final step, we screened variants residing within the 212 retinal disease-associated genes listed in the RetNet database that was last updated on March 10, 2014 (https://sph.uth.edu/retnet/).

### 2.3. Sanger Sequencing for* RHO* Mutations

Sanger sequencing for* RHO* mutations was conducted in two of the Japanese families, which included probands and other family members (Figures 1(a) and 1(b)). We used two primer pairs: a forward primer (RHO-2F), 5′-CTCCTCAAATCCCTCTCCCACTCCT-3′, and a reverse primer (RHO-2R), 5′-TCTTCTGCCCTACACCCCTACCCTG-3′ for exon 2, and a forward primer (RHO-5F), 5′-CGAACCTCACTAACGTGCCAG-3′, and a reverse primer (RHO-5R), 5′-GGTCTTGGTGGATGTCCCTTC-3′ for exon 5.

### 2.4. Molecular Modeling and Simulation

The models for molecular dynamics (MD) simulation were generated using two bovine rhodopsin/opsin crystal structures as the templates: the dark-adapted rhodopsin (Protein Data Bank ID: 1U19, chain A) and the ligand-free form opsin (Protein Data Bank ID: 3CAP, chain A). To examine the impact of the p.W126L mutation on protein conformation, 11-*cis*-retinal was removed from the dark-adapted template. Amino acids in these templates were replaced with the corresponding amino acids from the human sequence, thereby resulting in the wild-type (WT) models. The p.W126L models were generated from the WT models by changing the tryptophan at position 126 to leucine. All amino acid replacements were performed by using the simple mutate function of the Coot software [28]. The membrane environment was an artificially generated palmitoyl-oleoyl-phosphatidyl choline bilayer of approximately 120 molecules with a dimension of 80 × 80 Å. Each model was then merged with the membrane. All of the models we developed were superposed onto each other so that each model was embedded in the artificial membrane at almost identical orientations. The protein-membrane system was then solvated with water containing 150 mM NaCl. All manipulations were performed using Visual Molecular Dynamics version 1.9 [29].

MD simulations were run by the Not (just) Another Molecular Dynamics program [30] through the visual molecular dynamics interface using the following conditions: 1 fs per step, 100,000 steps (100 ps) for minimization, and 1,000,000 steps (1 ns), periodic boundary conditions and particle mesh Ewald method [31], cut-off at 10 Å, and switching at 9 Å. The calculations were carried out under a constant pressure and temperature ensemble at 310 K and 1 bar. A figure was prepared using the Discovery Studio Visualizer software (Accelrys Inc., San Diego, CA).

## 3. Results

### 3.1. Clinical Findings in Family 1 (JU#0678-062JIKEI)

#### 3.1.1. Patient II-2

**Figure 2:**
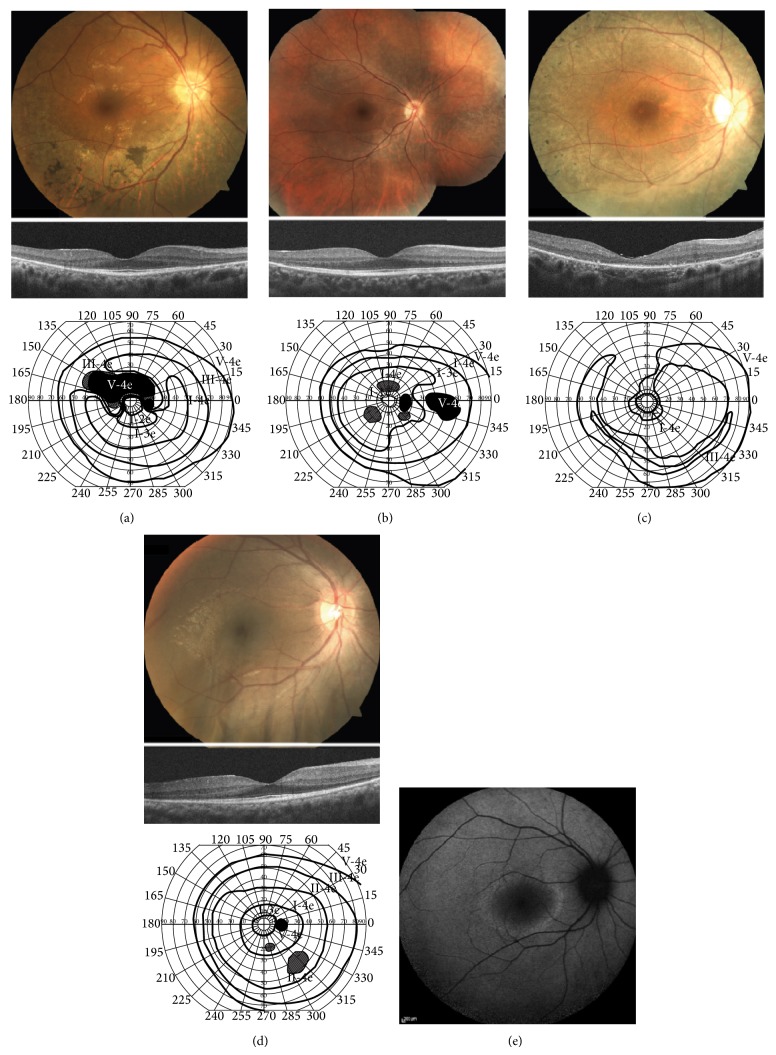
Fundus photographs, optical coherence tomography (OCT) images, and visual fields with Goldmann kinetic perimetry (GP) for the two Japanese families with retinitis pigmentosa. (a) Fundus photograph, OCT, and GP in the right eye of patient II-2 in family 1. (b) Fundus photograph, OCT, and GP in the right eye of patient III-1 in family 1. (c) Fundus photograph, OCT, and GP in the right eye of patient II-1 in family 2. (d) Fundus photograph, OCT, and GP in the right eye of patient III-1 in family 2. (e) A fundus autofluorescence image in the right eye of patient III-1 in family 2. See the Results section for details.

**Figure 3:**
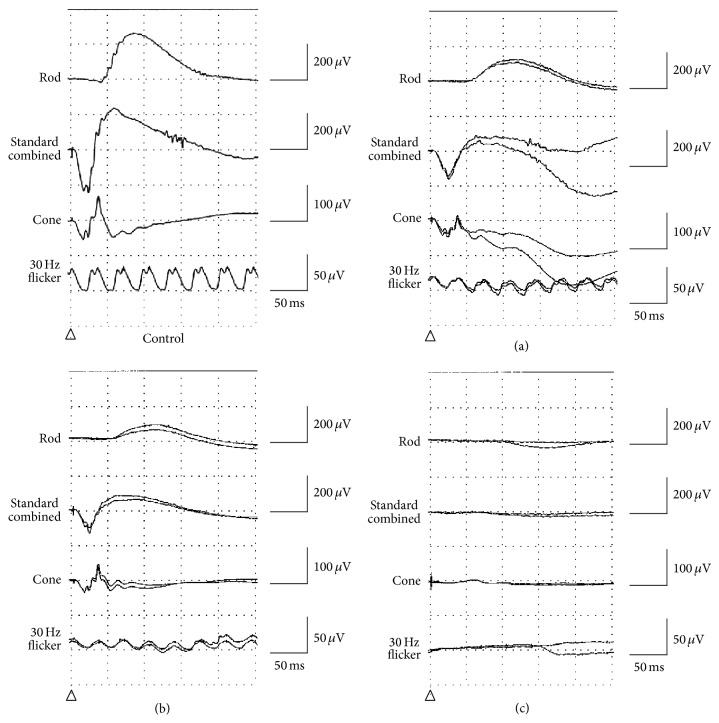
Full-field electroretinograms (ERGs). (a) and (b) ERG of both patient II-2 (a) and patient III-1 (b) in family 1 shows diminished amplitudes of the rod, standard combined, cone, and 30-Hz flicker responses. (c) ERG of patient II-1 in family 2 is nonrecordable for the rod, standard combined and 30-Hz flicker responses.

Patient II-2 (a proband) was a 58-year-old man, who was referred to our hospital because of suspicion of RP. BCVA at his first visit to our hospital was 1.5 (with +1.50 diopter (dpt), cylinder (cyl) −1.25 dpt axial (Ax) 20°) in his right eye and 0.7 (with +1.50 dpt., cyl. −0.50 dpt. Ax. 150°) in his left eye. No abnormalities were found except for slight senile cataracts in the anterior segments and media of both eyes. Intraocular pressures were within the normal range in both eyes. Fundus examination revealed retinal degeneration around the inferior vascular arcade in his right (Figure 2(a)) and left eyes. The OCT images showed marked thinning of the outer nuclear layer (ONL) and disruption of the inner segment/outer segment (IS/OS) line, except for the foveal region of his right (Figure 2(a)) and left eyes. GP at a previous hospital showed depression of visual fields and an arcuate scotoma at the superior visual field in his right (Figure 2(a)) and left eyes. Full-field ERG showed that there were decreased amplitudes in the rod, standard combined, cone, and 30-Hz flicker responses (Figure 3(a)).

#### 3.1.2. Patient III-1 (a Daughter of Patient II-2)

Patient III-1 was a 31-year-old woman, who reported night blindness. The clinical findings of patient III-1 were essentially similar to those found for patient II-2. BCVA at her first visit to our hospital was 1.5 (with cyl. −0.50 dpt. Ax. 160°) in her right eye and 1.2 (with cyl. −1.00 dpt. Ax. 35°) in her left eye. There were no abnormalities found in the anterior segments and media of either eye. Intraocular pressures were within the normal range in both eyes. Fundus examination revealed retinal degeneration in the nasal area of her right (Figure 2(b)) and left eyes. The OCT images showed marked thinning of the ONL and disruption of the IS/OS line except for the foveal region of her right (Figure 2(b)) and left eyes. GP showed several isolated scotomas in her right (Figure 2(b)) and left eyes. Full-field ERG showed that there were decreased amplitudes in the rod, standard combined, cone, and 30-Hz flicker responses (Figure 3(b)).

### 3.2. Clinical Findings in Family 2 (JU#0575-037JIKEI)

#### 3.2.1. Patient II-1

Patient II-1 (a proband) was a 35-year-old woman referred to our hospital for assessment of a causative gene for her RP. BCVA was 0.7 (with no correction) in her right eye and 0.6 (with cyl. −1.75 dpt. Ax. 30°) in her left eye. There were no abnormalities found in the anterior segments and media of either eye. Intraocular pressures were within the normal range in both eyes. Fundus examination revealed diffuse retinal degeneration and intraretinal pigment deposits with a bone-spicule configuration around the vascular arcade to the periphery of her right (Figure 2(c)) and left eyes. The OCT images showed marked thinning of the ONL and entire disruption of the IS/OS line in her right (Figure 2(c)) and left eyes. GP showed a ring-like defect in her right (Figure 2(c)) and left eyes. Full-field ERG showed that there were no responses in the rod, standard combined, and 30-Hz flicker ERG (Figure 3(c)).

#### 3.2.2. Patient III-1 (a Son of Patient II-1)

Patient III-1 was a 14-year-old boy, who was first found to have night blindness at 6 years of age. At the age of 11, a dark-adapted single flash ERG performed at another hospital showed that there were reduced responses in both eyes (data not shown). BCVA was 1.2 (with +6.50 dpt., cyl. −2.00 dpt. Ax. 180°) in his right eye and 1.0 (with +7.00 dpt., cyl. −1.50 dpt. Ax. 10°) in his left eye. Fundus examination revealed retinal degeneration of the inferotemporal areas in his right (Figure 2(d)) and left eyes. The OCT images showed that there was marked thinning of the ONL and disruption of the IS/OS line, except for the foveal region of his right (Figure 2(d)) and left eyes. GP showed constriction of visual fields with small scotomas in his right (Figure 2(d)) and left eyes. A fundus autofluorescence image revealed a perifoveal hyperautofluorescent ring in his right (Figure 2(e)) and left eyes. He was diagnosed as an early stage of RP.

### 3.3. Molecular Genetic Findings

The obtained sequence data were analyzed in accordance with the filtering steps discussed in the Material and Methods section.  Table 1 summarizes the remaining rare variants that were examined in families 1 and 2. The analysis focused on the 212 retinal disease-causing genes that were listed in the RetNet database. The two* RHO *mutations revealed in the data, c.377G>T, p.W126L in exon 2 and c.1036G>C, p.A346P in exon 5, were found in the adRP patients of families 1 and 2, respectively. With the exception of these* RHO* mutations, there were no other mutations found in the obtained sequence data for both families 1 and 2 that fulfilled the RP phenotype and autosomal dominant inheritance pattern conditions. The p.W126L mutation has not been previously reported in the literature and is not found in the dbSNP (http://www.ncbi.nlm.nih.gov/SNP/), 1000 Genomes database, or the HGMD (http://www.hgmd.org). On the other hand, the p.A346P mutation was previously reported to be the cause of adRP in one Spanish family [34]. We used* in silico* bioinformatics tools to investigate the impact of the p.W126L mutation on the RHO function. Results of the PolyPhen-2 program generated a score of 0.999 (probably damaging), which was close to the maximum value of 1.00, while the SIFT program generated a score of 0 (damaging).

For the cosegregation analysis, we investigated whether six of the family 1 members and four of the family 2 members had either the p.W126L or the p.A346P mutation. Our results revealed there was complete cosegregation of each mutation with the RP phenotype in each family (Figures 1(a) and 1(b)).

### 3.4. Molecular Modeling and Simulation

**Figure 4:**
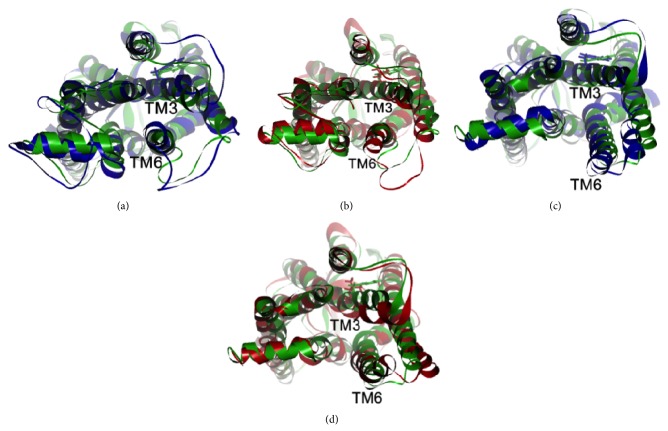
Projected view from the cytoplasmic side of the backbone before and after the 1 ns molecular dynamics (MD) simulations. The side chain of the position 126 in each model is shown by the bold sticks in the corresponding colors. (a) Dark-adapted like models. Blue: wild-type after 1 ns MD; green: wild-type before MD. (b) Dark-adapted like models. Red: W126L after 1 ns MD; green: W126L before MD. (c) Light-adapted like models. Blue: wild-type after 1 ns MD; green: wild-type before MD. (d) Light-adapted like models. Red: W126L after 1 ns MD; green: W126L before MD.

By using energy minimization and MD simulations, we investigated the possible effects of the p.W126L mutation on the structure of the human opsin moiety that was represented by either the dark-adapted like (high affinity for 11-*cis*-retinal) or the light-adapted like (high affinity for all-*trans*-retinal) crystal structures. Although the simulation time was limited, all four models examined approached similar levels of equilibria. Because the mutation site W126 is located in the third transmembrane helix (TM3), we analyzed differences in the TM3 backbone and in the nearby helices. In the dark-adapted template, the extracellular and central regions of TM3 did not appear to move significantly during the simulation regardless of the type of side chain (W or L) at position 126 (Figures 4(a) and 4(b)). This result corroborates the previous observation that the p.W126L mutation affected the 11-*cis*-retinal binding only marginally in COS-1 cells [35]. Interestingly, in the light-adapted like template, the TM3 helix within the heptahelical bundle moved differently in the WT and the p.W126L mutant models during the 1 ns simulation (Figures 4(c) and 4(d)). While the WT and the p.W126L mutant models exhibited only marginal changes in the TM3 during the first 300 ps of simulation (data not shown), this helix progressively tilted only in the p.W126L mutant during the subsequent 1 ns of simulation (Figure 4(d)). Persistence of this tilting during longer periods may lead to a decrease in the distance between the cytoplasmic ends of TM3 and TM6 and may affect the signal-transduction properties of the protein. The residue L125, which is next to W126 in bovine rhodopsin, is in contact with a highly conserved phenylalanine in the middle of the TM6 at residue 261. These side-chain interactions may therefore be critical for the ligand-induced activation of the rhodopsin-like G-protein-coupled receptors, similar to that which has been proposed for the *β*2-adrenergic receptor [36].

## 4. Discussion

In this study, we identified two* RHO* mutations (p.W126L and p.A346P) as disease causes by using whole-exome sequencing in two Japanese families with adRP. We additionally used molecular modeling to analyze the impact of the novel mutation (p.W126L) on the protein structure and function and then evaluated the genotype-phenotype correlations among Japanese RP patients with heterozygous* RHO* mutations.

For the cosegregation analysis, further validation by Sanger sequencing in other family members demonstrated there was complete cosegregation of each mutation with the RP phenotype (Figures 1(a) and 1(b)). For the novel p.W126L mutation, the tryptophan residue at the position 126 is located in the TM3 and is highly conserved among orthologs in vertebrates (Figure 1(c)). A biochemical experiment using reconstituted bovine rhodopsin has shown that an analog of 11-*cis*-retinal is cross-linked to the W126 residue [37]. Our* in silico* study, which used the PolyPhen-2 and SIFT programs, predicted that the p.W126L mutation would cause severe damage to the rhodopsin. In addition, the results of our protein modeling and simulations (Figure 4) suggest that the p.W126L mutation will likely affect the side-chain interaction between TM3 and TM6 in the light-adapted form but will not affect the interaction in the dark-adapted form. These molecular modeling and simulation findings suggest that the p.W126L mutation may impair rhodopsin function by affecting its conformational transition in the light-adapted form. This interpretation is in line with previous studies that have shown that p.W126L of bovine rhodopsin is less potent during G-protein activation [35]. Thus, these results predict that the p.W126L mutation can affect the RHO function, especially in the light-adapted form, thereby leading to the phenotype of RP. On the other hand, p.A346P has previously been reported to be an adRP-causing mutation in one Spanish family [34]. Both our cosegregation data for each family and the molecular modeling confirm that p.W126L is a disease-causing mutation.

With regard to the phenotypes of our patients, the p.W126L (family 1) and p.A346P (family 2) mutations are likely to be associated with sector RP and classic RP, respectively. However, it should be noted that patient III-1 (family 2) was not diagnosed with either classic or sector RP, as patient III-1 exhibited an early stage of RP. The phenotypes of patient II-1 (family 2) were similar to those previously reported for a patient of European descent who carried the p.A346P mutation and exhibited classic RP [34]. These findings suggest that the p.A346P mutation might be associated with the phenotype of classic RP.

When trying to diagnose classic or sector RP, it is highly important that one understands the severity and prognosis of* RHO*-associated adRP. As compared to classic RP, sector RP is considered to be a less severe disease with subnormal ERG and visual field defects that correspond to the affected sectors [13, 14]. In fact, it has been reported that sector RP is caused by a number of* RHO* mutations, including p.T4K [38], p.N15S [39–41], p.T17M [33, 42], p.P23H [10], p.T58R [12], p.N78I [43], and G106R [11, 25, 44]. To the best of our knowledge, there have been nine* RHO* mutations reported in the Japanese adRP population [20, 22–25, 32, 33, 41], with detailed phenotypes described in five (p.N15S, p.T17M, p.G106R, p.E181K, and p.P347L) out of the nine mutations [20, 23–25, 32, 33, 41]. Among these five* RHO *mutations, one (p.P347L) exhibited classic RP while the other four (p.N15S, p.T17M, p.G106R, and p.E181K) showed sector RP.  Table 2 summarizes the clinical features that were described for the genotype-phenotype correlations of the seven Japanese adRP families (including our families) with the seven* RHO* mutations (p.N15S, p.T17M, p.G106R, p.W126L, p.E181K, p.A346P, and p.P347L). Interestingly, patients with five other mutations (p.N15S [39–41], p.T17M [33, 42], p.G106R [11, 25, 44], p.A346P [34], and p.P347L [5, 32, 45]) were found to have phenotypes that were similar to Japanese and other ethnic groups, although the clinical phenotypes for two other mutations (p.W126L and p.E181K [20, 21, 46]) could not be sufficiently evaluated [5, 11, 20, 21, 25, 32–34, 39–42, 44–46]. These findings suggest that the location of each missense mutation is important for the purpose of predicting a diagnosis of either classic or sector RP and that there is a similarity of phenotypes between Japanese and other ethnic group RP patients who have identical* RHO* mutations. Also Sandberg et al. report the phenotype-genotype correlations between adRP patients with* RHO* mutations, revealing that patients with mutations altering the intradiscal domain near the N-terminal region (or a low-numbered codon) tended to have better visual function than patients with mutations altering the cytoplasmic domain near the C-terminal region (or a high numbered codon); patients with a mutation altering the transmembrane domain or a mid-numbered codon had intermediate function [47]. Thus, identification of* RHO* mutations appears to be useful for predicting the severity of the RP phenotypes and providing precise genetic counseling.

In conclusion, we identified two* RHO* mutations (p.W126L and p.A346P) in two Japanese families with adRP. The p.W126L mutation has not been previously reported in any ethnic groups. The genotype-phenotype correlations indicated that the location of the* RHO* mutations is likely to determine the phenotype of either classic or sector RP. Identification of* RHO *mutations is a very useful tool for predicting the disease severity and providing precise genetic counseling.

## Figures and Tables

**Table 1:** The rare variants found in the two Japanese families with *RHO* mutations, focusing on 212 retinal disease-causing genes registered in the RetNet database (https://sph.uth.edu/retnet/).

JU0678-062JIKEI		Gene	Gene Bank ID	Exon	Nucleotide change	AA change	State	SNP ID
Chrom	Position							
2	202498104	*TMEM237 *	NM_001044385	5	c.325C>T	p.R109X	Hetero	
3	129249734	*RHO *	NM_000539	2	c.377G>T	p.W126L	Hetero	
4	6290790	*WFS1 *	NM_006005	4	c.392T>G	p.V131G	Hetero	
4	6302786	*WFS1 *	NM_006005	8	c.1264G>T	p.A422S	Hetero	
7	33427676	*BBS9 *	NM_198428	19	c.2035C>T	p.R679W	Hetero	
8	10480476	*RP1L1 *	NM_178857	2	c.236G>A	p.R79H	Hetero	
14	21792816	*RPGRIP1 *	NM_020366	14	c.1802C>T	p.S601L	Hetero	rs3748360
16	49670817	*ZNF423 *	NM_015069	4	c.2243_2245del	p.748_749del	Hetero	

JU0575-037JIKEI		Gene	Gene Bank ID	Exon	Nucleotide change	AA change	State	SNP ID
Chrom	Position							

1	94476477	*ABCA4 *	NM_000350	40	c.5593C>T	p.H1865Y	Hetero	rs201707267
2	112751865	*MERTK *	NM_006343	9	c.1334G>A	p.R445Q	Hetero	rs202242962
3	129252550	*RHO *	NM_000539	5	c.1036G>C	p.A346P	Hetero	
11	17531103	*USH1C *	NM_153676	18	c.1813A>C	p.I605L	Hetero	
16	16291933	*ABCC6 *	NM_001171	10	c.1283A>G	p.N428S	Hetero	rs201880691

Chrom = choromosome, AA = amino acid, Homo = homozygous, and Hetero = heterozygous.

**Table 2:** Clinical summary of Japanese patients with autosomal dominant retinitis pigmentosa with heterozygous *RHO* mutations.

Patient, gender	Type of adRP	Age at examination	Mutation	BCVA		Electroretinograms (ERGs)		Reference	Notes
				R	L	Flash (rod plus cone) ERG	Full-field ERG		
Case 1, M	Classic	44	p.P347L	0.5	0.66	NR	NR in 30-Hz flicker	[23, 32]	Cataract
Case 2, F	Classic	20	p.P347L	1.0	1.0	NR	NR in 30-Hz flicker	[23, 32]	
Case 3, F	ND	11	p.P347L	1.0	1.0	Reduced	Reduced in 30-Hz flicker	[23, 32]	
Case 4, F	Classic	75	p.P347L	LP	LP	NR	NR in 30-Hz flicker	[23, 32]	Severe cataract
III-6, M	Sector	49	p.T17M	0.2	0.2	ND	Reduced in both rods and cones	[23, 33]	B-CME, L-CNV
Proband, F	Classic	39	p.E181K	0.1	0.1	ND	NR in rods, reduced in cones	[20]	B-CME
III-5, F	Sector	52	p.N15S	ND	ND	ND	ND	[24]	
II-2, M	Sector	66	p.G106R	0.04	0.5	ND	Reduced in both rods and cones	[25]	R-CME
III-1, F	Sector	44	p.G106R	1.2	1.2	ND	Reduced in rods, normal in cones	[25]	
III-2, F	Sector	40	p.G106R	1.2	1.2	ND	Reduced in rods, normal in cones	[25]	
II-2 (FN.1), M	Sector	58	p.W126L	1.5	0.7	ND	Reduced in both rods and cones	Current study	
III-1 (FN.1), F	Sector	31	p.W126L	1.5	1.2	ND	Reduced in both rods and cones	Current study	
II-1 (FN.2), F	Classic	35	p.A346P	0.7	0.6	ND	NR in both rods and cones	Current study	
III-1 (FN.2), M	ND	14	p.A346P	1.2	1.5	Reduced	ND	Current study	

BCVA = decimal best-corrected visual acuity; R = right eye; L = left eye; B = both eyes; M = male; F = female; FN = family number; ND = not described or not done; NR = nonrecordable; LP = light perception; CNV = choroidal neovascularization; and CME = cystoid macular edema.
